# Identifying functional subtypes and common mechanisms of rheumatoid arthritis and systemic lupus erythematosus

**DOI:** 10.1016/j.gendis.2025.101527

**Published:** 2025-01-10

**Authors:** Jiajun Li, Hao Tang, Zhenwei Shang, Rui Chen, Xin Meng, Xiangshu Cheng, Zerun Song, Shuai Li, Ruijie Zhang, Wenhua Lv

**Affiliations:** College of Bioinformatics Science and Technology, Harbin Medical University, Harbin, Helongjiang 150086, China

There are similarities between rheumatoid arthritis (RA) and systemic lupus erythematosus (SLE) in terms of clinical manifestations, immune responses, and therapeutic strategies,^1^ and thus a joint analysis of the two diseases could contribute to a deeper understanding of the shared pathogenesis of autoimmune diseases. The subtype analysis of RA and SLE is currently understudied, and the marker genes used for subtype definition in most studies are derived from bulk RNA sequencing data or microarray data, which are underrepresentative of individual immune cell status.^2^ Therefore, we aimed to identify cell type-specific expressed genes as biomarkers based on single-cell RNA sequencing data and to explore the commonalities and differences between RA and SLE by a combined subtype analysis based on microarray data. Both the representativeness of the markers in terms of immune characteristics and the reproducibility of the results are ensured by the sufficient sample size. Immune infiltration analysis revealed the subtype heterogeneity and significant differences in clinical characteristics between different subtypes of RA patients, which verified the heterogeneity between different subtypes. Finally, we constructed subtype prediction models by machine learning algorithms further validating the heterogeneity among subtypes. Detailed methodology and the overall flowchart (Fig. S1) are provided in the supplementary material.

Single-cell RNA sequencing data from peripheral blood mononuclear cells of RA patients, SLE patients, and healthy controls (GSE159117 and GSE162577) were collected, and cell clustering was performed, resulting in seven cell types including T cells, natural killer cells, B cells, monocytes, neutrophils, megakaryocytes, and dendritic cells (Fig. 1A; Fig. S2). In the disease group, B cell and monocyte proportions increased from 5.9% to 13.0% and 12.9%–25.9%, respectively, while T cell proportion decreased from 70.7% to 48.2%, highlighting the importance of these cell types in disease pathology (Fig. 1B and Table S1). Then, the top 138 cell-specific genes for T cells, B cells, and monocytes were selected as the marker genes for subtype analysis (Table S2). Two subtypes of RA and SLE samples (GSE50772 and GSE61635) were defined based on consensus clustering where two subtype groups consisted of 149 and 243 samples, respectively (Table S3). Interestingly, both subtype groups contained RA and SLE individuals, indicating the potential shared functional mechanisms between RA and SLE. The expression distribution of 138 signature genes had distinct expression patterns in different cell types for the two subgroups (Fig. 1C). The robustness of signature genes and subgroup definition was validated by external datasets (Supplementary File; Fig. S3 and Table S4–7).

**Figure 1:**
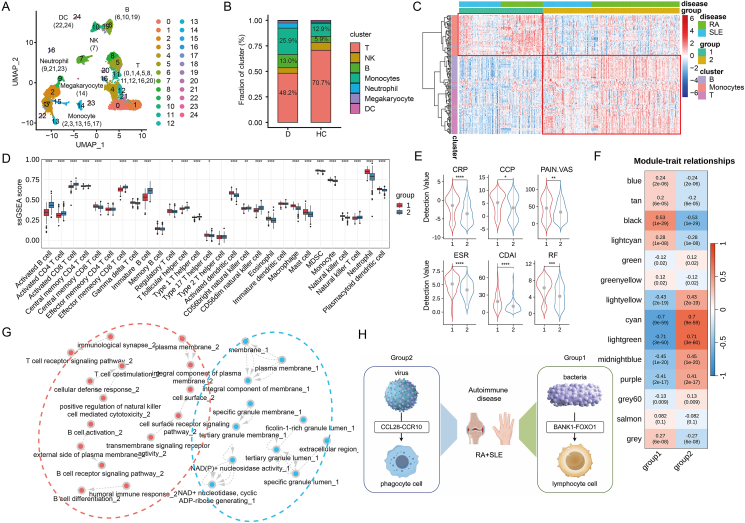
Key findings of this research. **(A)** A total of 25 cell clusters were divided into 7 different cell types and distinguished by different colors. **(B)** The percentage stacked bar chart shows the percentage of different cell types in the total number of cells in the control group and the disease group. **(C)** The heat map shows the expression differences of different cell cluster marker genes in different cluster populations. **(D)** ssGSEA algorithm was used to determine the differences in immune infiltration levels among different subtypes. ns, *P* > 0.05; ∗*P* ≤ 0.05, ∗∗*P* ≤ 0.01, ∗∗∗*P* ≤ 0.001, and ∗∗∗∗*P* ≤ 0.0001. **(E)** The violin chart shows the differences in clinical indicators among patients with different subtypes of rheumatoid arthritis. **(F)** The correlation between different modules and subtypes. The numbers in each cell represent correlation and significance. **(G)** The network diagram shows the correlation between functional pathways. Among them, red and blue represent pathways enriched by two subtypes. **(H)** Hypothetical mechanisms underlying the two subtypes.

To confirm the reliability of shared subtypes between RA and SLE, we explored the heterogeneity between different disease subtypes. It showed significant differences in immune cell infiltration between disease subtypes. Specifically, for subtype Ⅰ, the immune infiltration degree of phagocytic cells, including macrophages, monocytes, mast cells, dendritic cells, neutrophils, and eosinophils was higher than that of subtype Ⅱ. For subtype Ⅱ, most lymphocytes including various T cells, B cells, and natural killer cells had a higher immune infiltration level than that of subtype Ⅰ, consistent with the pattern revealed by subtype classification (Fig. 1D).

To further investigate the subtype differences and to validate the reliability of the classification, we analyzed six clinical characteristics including C-reactive protein, anti-cyclic citrulline peptide, the visual analogue scale for pain, erythrocyte sedimentation rate, clinical disease activity index, and rheumatoid factor for 232 RA patients reflecting the disease severity. Significant differences for all six indicators between different RA subtypes are observed (*P* < 0.05; Fig. 1E). Moreover, compared with subtype Ⅱ, RA patients of subtype Ⅰ have higher disease severity.

The weighted gene co-expression network analysis (WGCNA) method was employed to identify co-expression modules associated with the disease subtypes (Fig. S4) and three subtype-correlated modules were identified. The black module had a correlation coefficient of 0.53 with subtype Ⅰ, while the turquoise and light green modules had a correlation coefficient of 0.71 and 0.70 with subtype Ⅱ, respectively (*P* < 0.001; Fig. 1F; Fig. S5A–C). Subtype Ⅰ related module is mainly involved in functions including phagocytosis, activation of immune cells, positive regulation of inflammatory response, and defense response to bacteria (Fig. S5D–F). Therefore, we hypothesized the mechanism of disease subtype Ⅰ as follows: a patient has immune responses leading to abnormal activation of phagocytes (*e.g.*, macrophages and neutrophils) after a bacterial infection, which ultimately leads to the development of an immune disease. The subtype Ⅱ related module is mainly involved in functions associated with T cell activation, differentiation, T-cell receptor, B cell activation, and B-cell receptor signaling pathway, indicating that the biological mechanism of subtype Ⅱ is closely related to the activation of lymphocytes.

Furthermore, 302 up-regulated genes and 138 down-regulated genes were identified between subtype Ⅱ and subtype Ⅰ (Table S8). For subtype Ⅰ, down-regulated genes were enriched in terms related to inflammatory response and defense against bacteria, validating our previous hypotheses about the biological mechanism of subtype Ⅰ. For subtype Ⅱ, up-regulated genes were enriched in T and B cell activation and differentiation, consistent with the functional enrichment results of the WGCNA co-expression module. In addition, we discovered genes that were related to the positive regulation of natural killer cell-mediated cytotoxicity in the characteristic genes of subtype Ⅱ. Positive regulation of natural killer cell-mediated cytotoxicity refers to the enhancement of natural killer cell's killing effect on infectious pathogens through a series of signaling pathways and factors, while cytotoxicity refers to the phenomenon of viral infection leading to host cell death or damage (Fig. S6A–C). This implies that viruses may be the source of pathogenesis in subtype Ⅱ. Specifically, viral infection leads to abnormal activation of lymphocytes, which may cause immune dysregulation. In conclusion, subtype Ⅰ may be caused by bacterial infection, leading to abnormal activation of phagocytic cells, while subtype Ⅱ may be caused by viral infection, leading to abnormal activation of lymphocytic cells. Then, the gene set enrichment analysis (GSEA) was performed using immune function datasets from the MsigDB database as background gene sets. Significantly enriched pathways included phagocytic cells and lymphocytic cells, further validating the previous biological functional mechanism inference (Fig. S6D–F). The functional correlations between different groups were demonstrated by the network map (Fig. 1G).

To further investigate the underlying mechanisms of the subtypes and facilitate disease diagnosis and treatment, 50 potential gene biomarkers were selected from 440 differentially expressed genes by LASSO dimensionality reduction. The top 10 markers achieved strong classification performance with an average AUC of 0.993 (Fig. S7, 8). Notably, signaling lymphocytic activation molecule family member 6 (SLAMF6), C–C motif chemokine ligand 28 (CCL28), and B cell scaffold protein with ankyrin repeats 1 (BANK1)^3^, ^4^, ^5^ have been reported to be involved in immune response or to be related to autoimmune diseases (Supplementary File). Therefore, we speculate that a potential pathway influencing subtype Ⅰ may involve bacterial infection, leading to BANK1-FOXO1 (forkhead box O1) pathway dysregulation and lymphocyte abnormalities. Conversely, subtype Ⅱ may be impacted by viral factors affecting the CCL28-CCR10 (C–C motif chemokine receptor 10) pathway, resulting in phagocyte-related abnormalities (Fig. 1H).

In summary, we aim to identify shared subtypes between RA and SLE, whereas previous classification studies have focused on a single disease. This joint analysis explores similarities and differences between different diseases, which helps to better identify consistent targets for drug treatment, further classify diseases and guide treatment plans for patients. Besides, we innovatively selected cell-type-specifically expressed genes obtained from single-cell RNA sequencing data as signature genes for sample clustering, rather than using differentially expressed genes derived from microarray or bulk RNA sequencing data, which accurately differentiate between different cell types and therefore accurately perform disease clustering and classification. This study may provide new perspectives for the analysis of subtypes and molecular mechanisms of autoimmune diseases.

## Funding

This work was supported by the Fundamental Research Funds for the Provincial Universities in Heilongjiang Province, China (2024, to Wenhua Lv) and College Student Innovation Training Project of Heilongjiang Province, China (S202410226008).

## CRediT authorship contribution statement

**Jiajun Li:** Formal analysis, Methodology, Writing – original draft. **Hao Tang:** Data curation, Investigation, Visualization. **Zhenwei Shang:** Formal analysis, Resources, Software. **Rui Chen:** Data curation, Visualization. **Xin Meng:** Investigation, Validation. **Xiangshu Cheng:** Software, Visualization. **Zerun Song:** Resources, Software. **Shuai Li:** Investigation, Visualization. **Ruijie Zhang:** Conceptualization, Supervision, Writing – review & editing. **Wenhua Lv:** Conceptualization, Supervision, Validation, Writing – review & editing.

## Conflict of interests

The authors have no competing interests to declare.
