# Supplementary material for: Identifying functional subtypes and common mechanisms of rheumatoid arthritis and systemic lupus erythematosus
Source: Genes Dis. 2025 Jan 10;12(5):101527. doi: 10.1016/j.gendis.2025.101527 (PMC12166693; doi:10.1016/j.gendis.2025.101527)
Supplement: Multimedia component 1 [file mmc1.docx]

**Supplmentary materials and methods**

Data collection

The dataset used in this study was obtained from the GEO database. The scRNA-seq dataset with 10X Genomics platform included peripheral blood mononuclear cells (PBMCs) from one RA patient (GSE159117) and one SLE patient (GSM4954811) and one healthy control (GSM4954813) form GSE162577. The validation dataset included single-cell PBMC sequencing data from three SLE samples in GSE142016, along with data from 18 RA patients and 18 healthy controls provided by Marina Sirota et al [[1](#_ENREF_1)]. To ensure accurate results, all datasets were subjected to the same sample processing procedures, avoiding the influence of other factors on the research results.

The microarray data included three datasets, consisting of SLE data from GSE50772 with 61 SLE patients and 20 healthy controls and GSE61635 with 99 SLE patients and 30 healthy controls, and RA data from GSE93272 with 232 RA patients and 43 healthy controls. It is worth noting that all three datasets were sequenced on the GPL570 platform, and all of the sequencing samples were blood samples thus ensuring the accuracy of the results.

ScRNA sequencing data processing

Single-cell RNA sequencing (scRNA-seq) data were analyzed by Seurat R package[[2](#_ENREF_2)]. For cell filtering, cells with gene numbers below 200 or above 4000, as well as cells with mitochondrial gene numbers exceeding 10% of the total cells were excluded. The FindIntegrationAnchors function from the Seurat package was used to remove batch effects between samples, as evidenced by the **Figure S9** showing effective batch correction. The FindClusters function was used to cluster cells based on the similarity of co-expressed genes, and the dataset resolution was set to 0.9. The FindAllMarks function was used to identify differentially expressed genes (DEGs) for each cluster, with a logfc.threshold of 0.25. Cell type identification was performed by comparing DEGs in each cluster to known marker genes for specific cell types, and manual verification was conducted using the cellMaker database[[3](#_ENREF_3)].

Microarray data processing

The Affy R package was used to process data downloaded from the GEO database (_ROW.tar)[[4](#_ENREF_4)]. To eliminate batch effects, the ComBat function in the sva package was used to merge the microarray data from both sources[[5](#_ENREF_5)](Figure S1).

Subtype classification based on Consensus Clustering

In order to determine the optimal number of clusters and biomarkers, the "ConsensusClusterPlus" R package was used for consensus clustering[[6](#_ENREF_6)]. In terms of the clustering algorithm,the K-means algorithm and the Euclidean distance were used as the distance metric. 500 iterations on 80% of the samples were performed to obtain classification results for different numbers of clusters.

Robustness verification

To control for disease-specific differences potentially caused by cell number variations across samples, we randomly selected three alternative SLE samples (GSE142016) to replace the original SLE sample for validation. Cell-type-specific genes were calculated, and consistent results were obtained across all trials, with overlap rates of marker genes reaching 80%, 78%, and 92%, respectively (Figure S3A-C). For further validation of clustering stability, we paired five SLE samples (GSE162577 and GSE142016) with five randomly selected RA patients and five healthy controls. Single-cell analysis was performed to identify cell-type-specific marker genes, which were subsequently used for subtype clustering of RA and SLE samples. Among 393 individuals, the clustering results were consistent in over 92% of cases, confirming the robustness of the identified subtypes across both disease groups (Figure S3D).

Evaluation of the composition of immune microenvironment

The single-sample gene set enrichment analysis (ssGSEA) method were utilized to assess the enrichment level of immune gene sets in individual samples[[7](#_ENREF_7)]. This gene set includes 782 genes corresponding to 28 types of immune cells, as reported by Pornpimol Charoentong et al[[8](#_ENREF_8)]. Through this approach, the immune-related functions of individual samples were revealed.

CIBERSORT, a gene expression-based immune infiltration analysis method, was used to assess the proportions and quantities of different immune cell types in tumor tissues[[9](#_ENREF_9)]. The gene expression signature matrix of 22 infiltrating immune cells provided by the CIBERSORT platform (https://Cibersortx.stanford.edu/) were utilized to obtain the infiltration proportions of the 22 immune cell types.

Weighted gene co-expression network for identifying subtype related modules

Weighted Gene Co-Expression Network Analysis (WGCNA) and identification of gene expression patterns and functional correlations were conducted by the WGCNA package in R software[[10](#_ENREF_10)]. The top 5000 genes with highest variance were selected to perform clustering using hierarchical clustering algorithm. Subsequently, the clustering results were divided into multiple modules by using Dynamic Tree Cut algorithm.

Identification of differentially expressed genes

To calculate differential expressed genes between different subtypes, the limma package was used and significantly dysregulated genes were defined as genes with corrected p-values less than 0.05 and absolute logFC greater than 0.5[[11](#_ENREF_11)].

Functional enrichment analysis to explore functional mechanisms of subtypes

We performed Gene Ontology (GO) and pathway enrichment analysis using the clusterProfiler and enrichplot R packages[[12](#_ENREF_12)]. Visualization of the enrichment results was conducted by the dotplot function from enrichplot package. We also calculated the overlap proportion of significant genes between pairwise functional gene sets and considered a proportion of greater than or equal to 0.3 as a potential correlation between the two functional sets. The functional network interaction diagrams were used to connect these related pathways and GO terms.

In the GSEA analysis, the immune function dataset (c7.v2023.1) from the MsigDB database was treated as the background gene set or biological pathway[[13](#_ENREF_13)]. GSEA analysis was implemented with the GSVA package, where the enrichment score (ES) represents the degree of significant enrichment of a gene set in the gene expression profile, with larger ES values indicating higher enrichment degree. To reliably compare the enrichment levels between different gene sets, we used the normalized enrichment score (NES) for further comparison analysis, which is obtained by dividing the ES value by the average ES value obtained from random permutations of the gene set[[14](#_ENREF_14)].

Construction of subtype classifier based on random forest and LASSO regression model

LASSO is a regression analysis method that requires standardization of the data to avoid the influence of different features[[15](#_ENREF_15)]. The data was standardize by scale function, and then the LASSO regression model was constructed by glmnet function in the glmnet R package.

We built a classifier model by randomForest function in the randomForest R package and defined the number of decision trees as 500[[16](#_ENREF_16)]. The genes were ranked by information gain, and the error was calculated for different numbers of genes using the random perturbation method. The appropriate number of genes was chosen to construct the final random forest model, and the predict function was used for prediction.

Statistical analysis

T-tests was performed to compare the differences between two groups of samples. It was considered statistically significant when the p-value was less than 0.05. We used the stat_compare_means function in the ggplot2 R package for T-test analysis and labeled the p-values less than 0.05 with *.

**2. Supplmentary Figures**

**
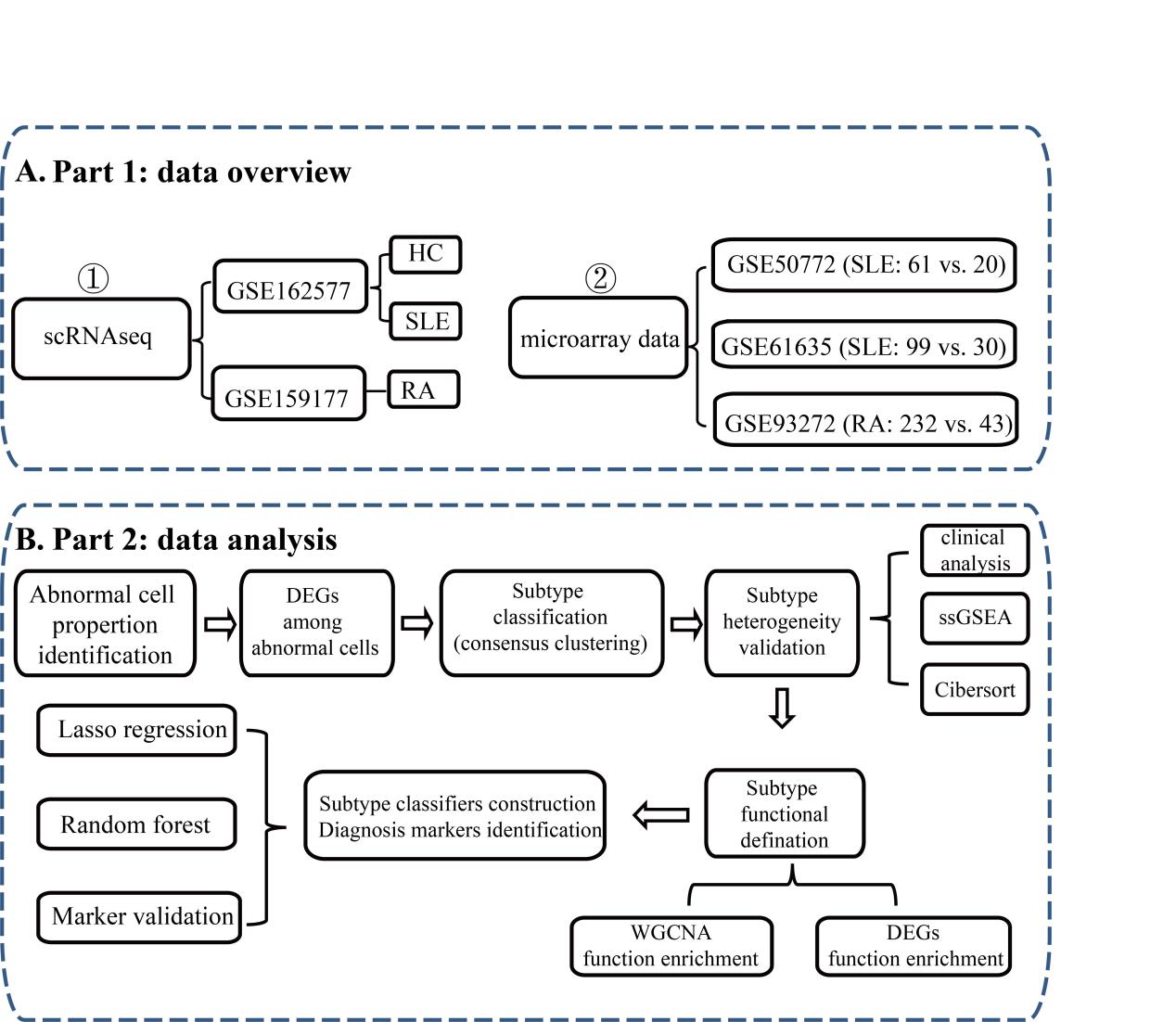
**

**Figure S1. The overall technology roadmap for this study.** A. Data overview. B. Data analysis process.


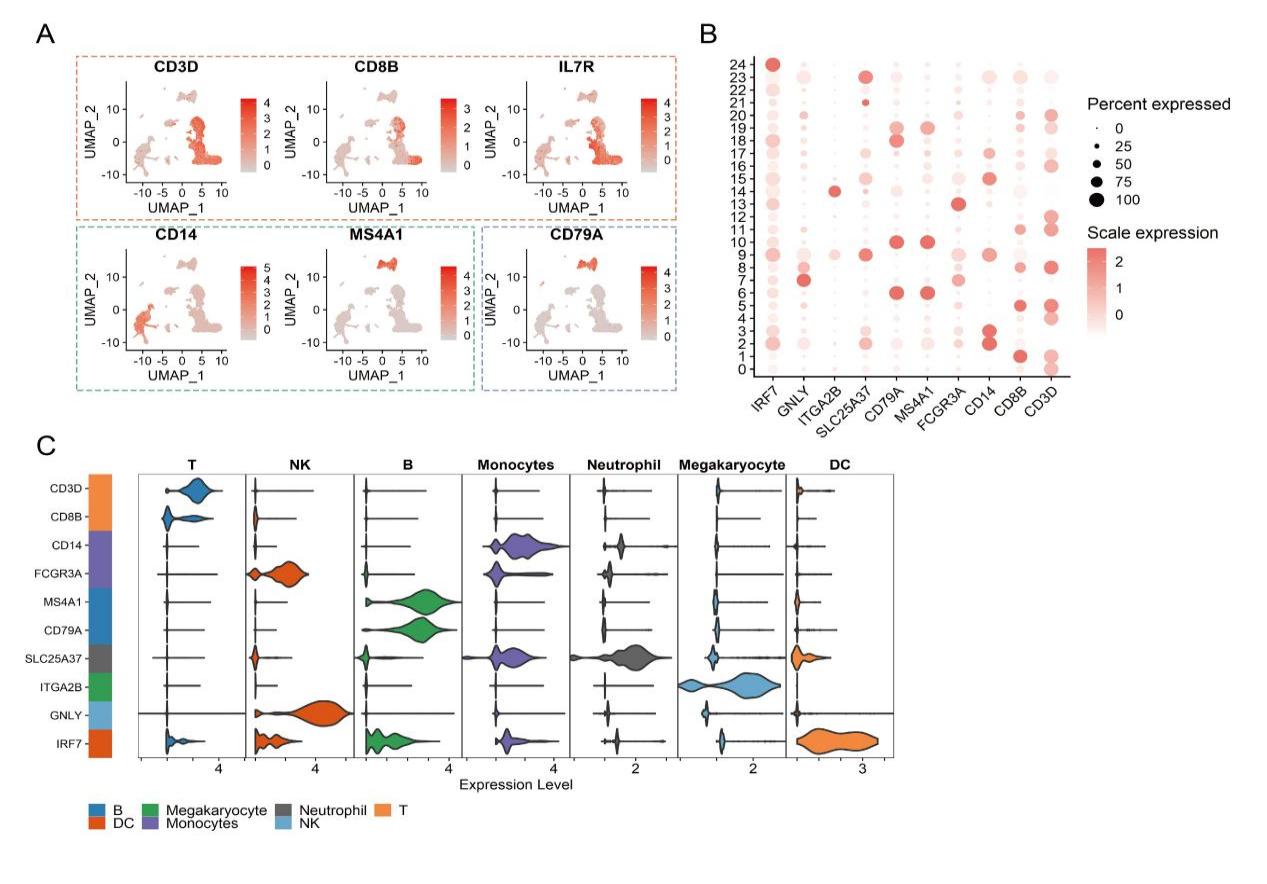


**Figure S2. Cell clustering and annotation in scRNA seq.** (A) The UMAP diagram shows the expression distribution of six biomarkers; (B) The heat map displays the expression of marker genes in different cell clusters; (C) The violin diagram shows the expression differences of markers in different cell types.


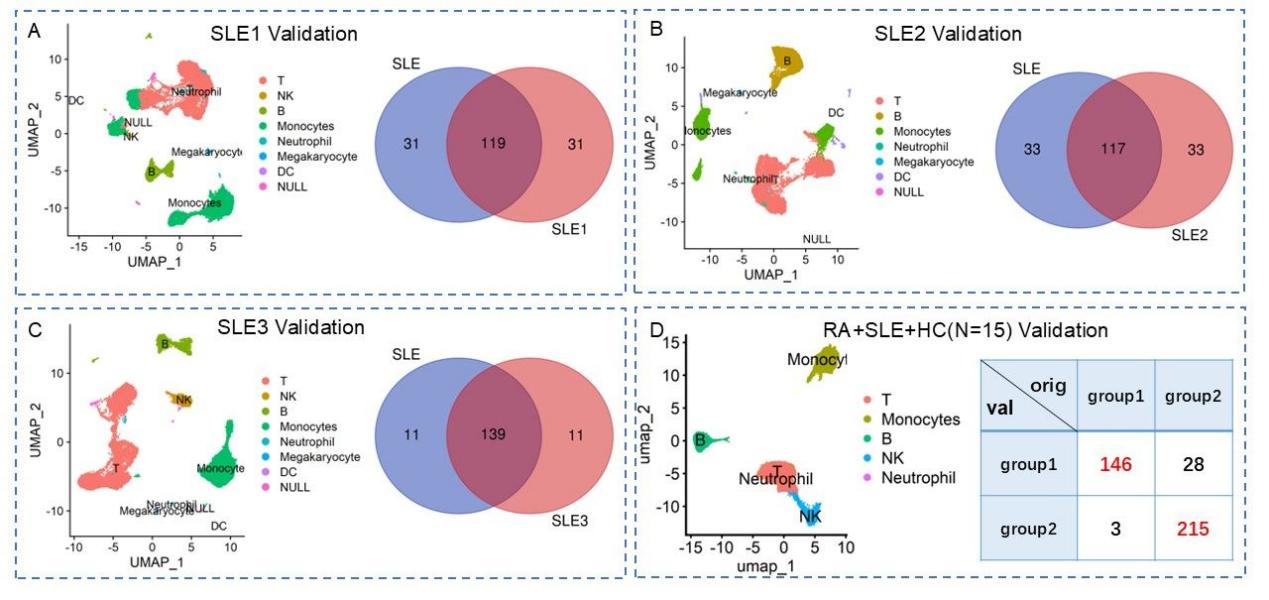


**Figure S3. Stability Assessment of scRNA Analysis Results with Different Samples.** (A–C) demonstrate the stability of cell-type-specific gene identification by replacing the original SLE dataset with different SLE samples (SLE1, SLE2, and SLE3). The Venn diagrams show the overlap of cell-type-specific genes between each substituted sample and the original dataset, indicating consistent gene identification across different samples. (D) evaluates the clustering consistency across 15 samples (RA, SLE, and HC) compared to the original clustering; the table displays the number of samples consistently clustered in both analyses**.**


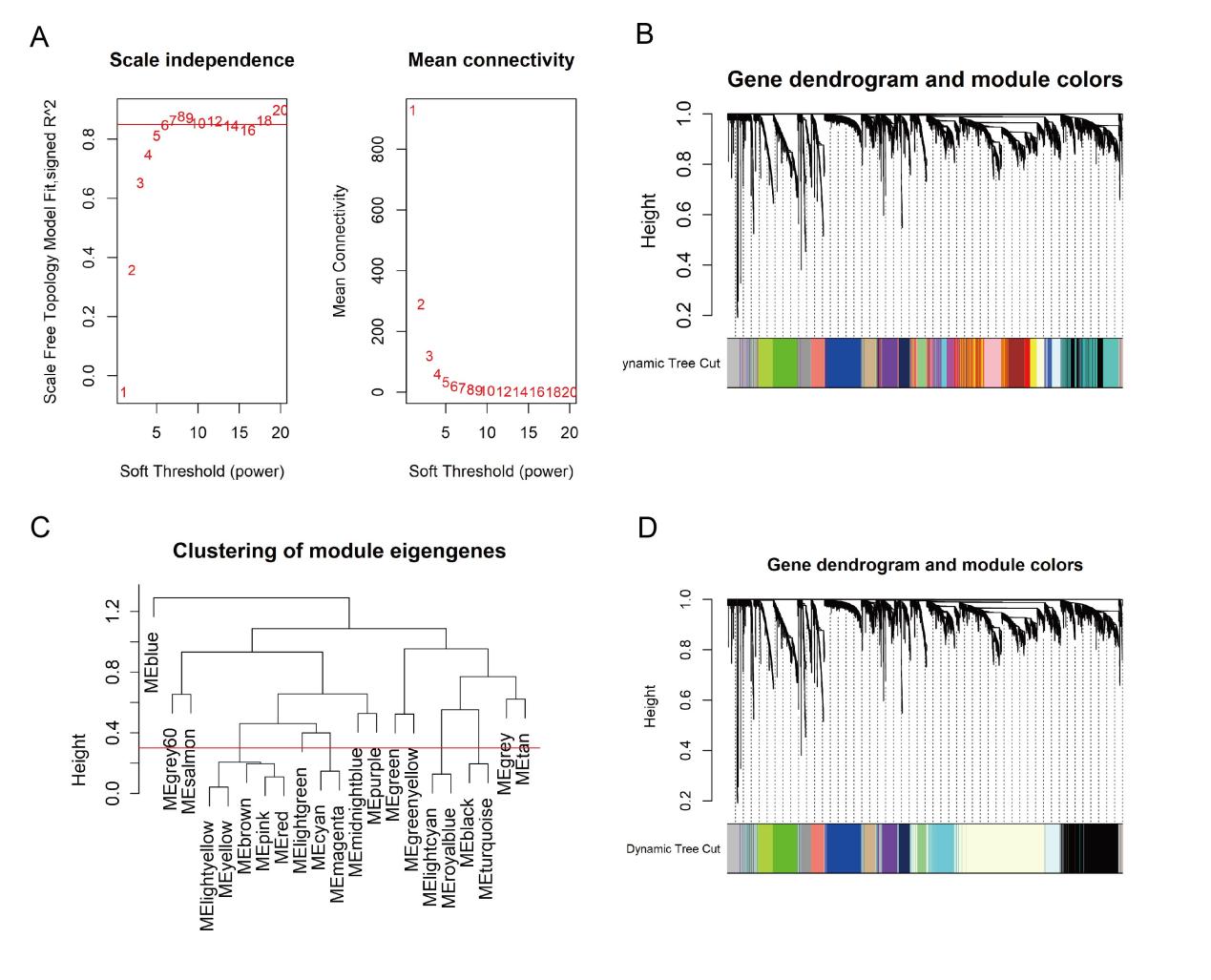


**Figure S4. WGCNA algorithm recognition co expression module.** (A) The left figure shows the scale-free fitting index under different soft thresholds, while the right figure shows the network connectivity under different soft thresholds; (B) Divide genes into different co-expression modules; (C) Score the similarity of different modules with a height threshold of 0.3, and merge the modules with high similarity; (D) Display the merged modules and their corresponding colors.


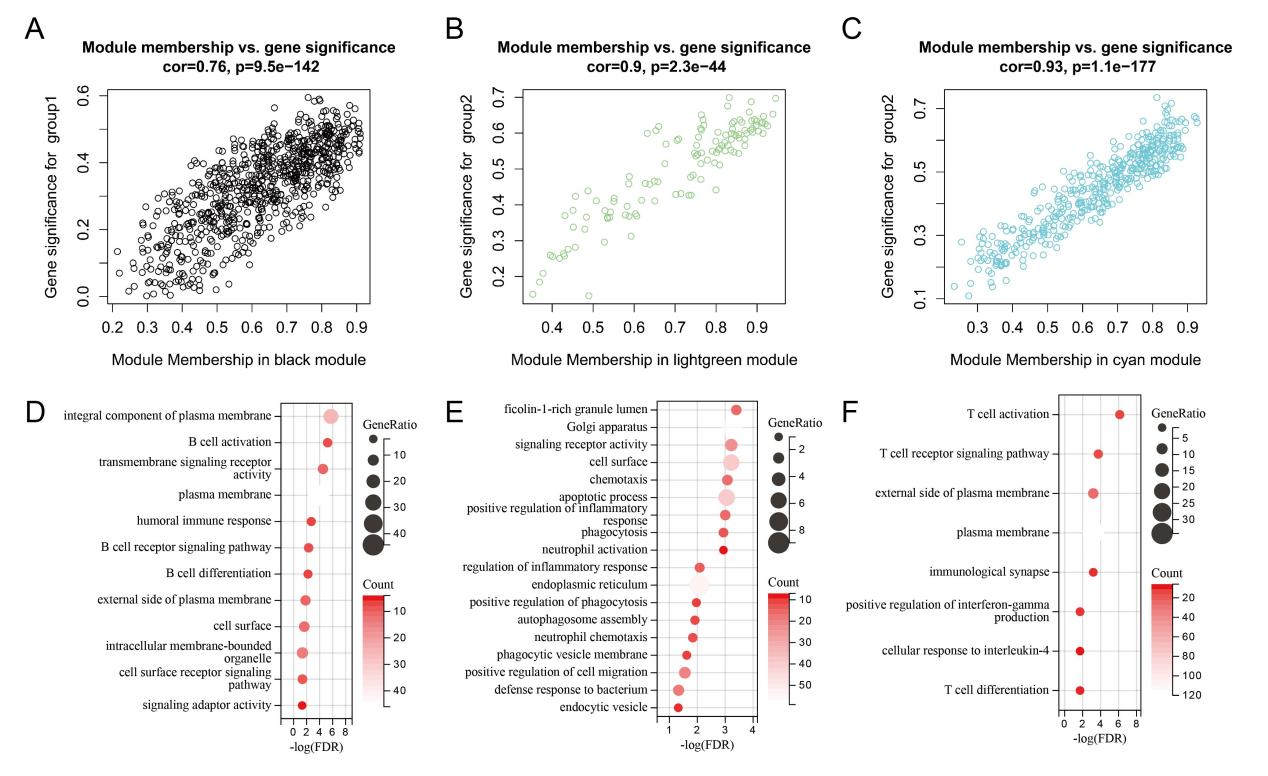


**Figure S5. The results of correlation and GO functional enrichment analysis within the co-expression module.** (A,B,C) Scatter plots of module feature genes for the three modules, represented by black, light green, and cyan respectively; (D,E,F) The results of GO functional enrichment of module characteristic genes in three modules.


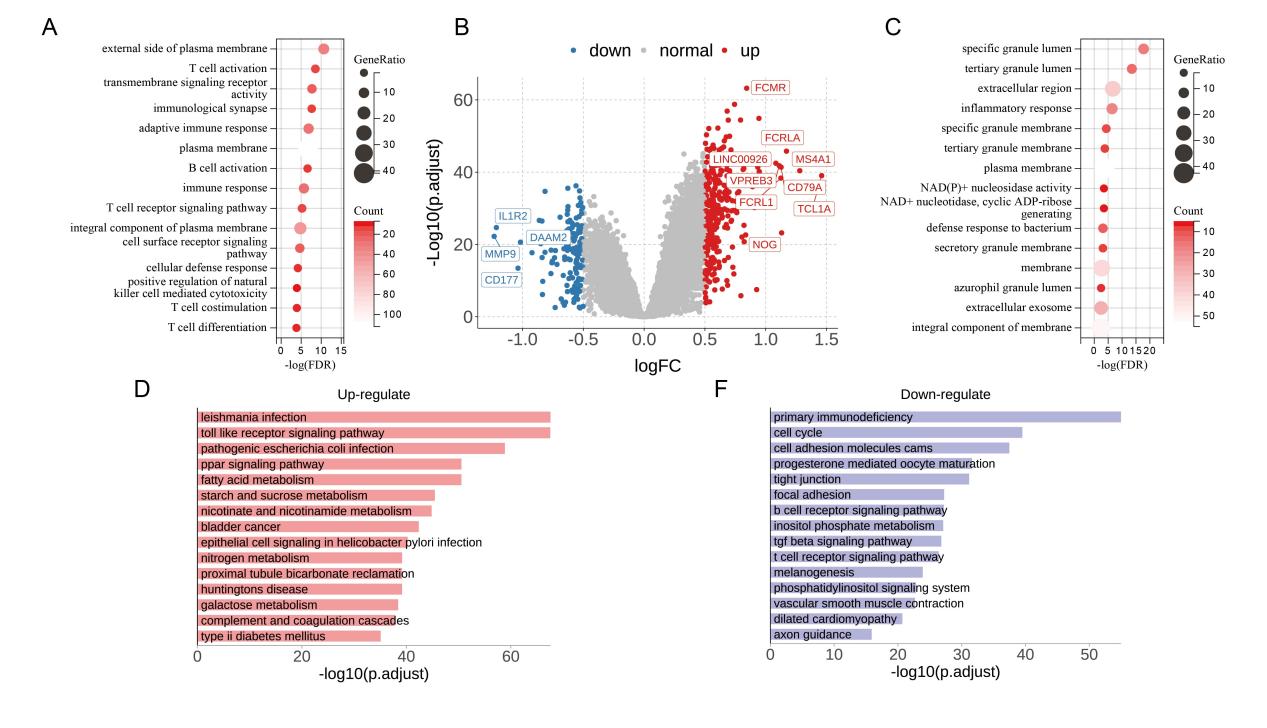


**Figure S6. The results of correlation and GO functional enrichment analysis within the co-expression module.** (A,B,C) Scatter plots of module feature genes for the three modules, represented by black, light green, and cyan respectively; (D,F) The results of GO functional enrichment of module characteristic genes in three modules.


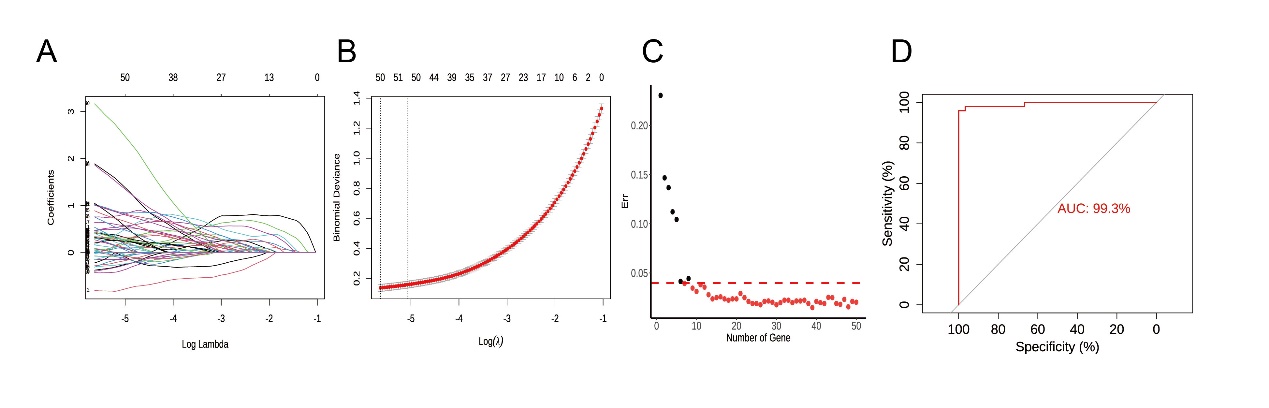


**Figure S7. LASSO regression and construction of random forest model.** (A) Each line in the graph represents the coefficient of a gene in the LASSO regression model, and the ordinate represents the coefficient size of the gene; (B) Different λ The error value of the LASSO regression model under the value, represented by the horizontal axis λ Different values of; (C) The average error of Random forest model under different gene numbers. The abscissa is the gene number, and the ordinate is the model error; (D) The Receiver operating characteristic shows the performance of the model, and AUC represents the area under the curve.


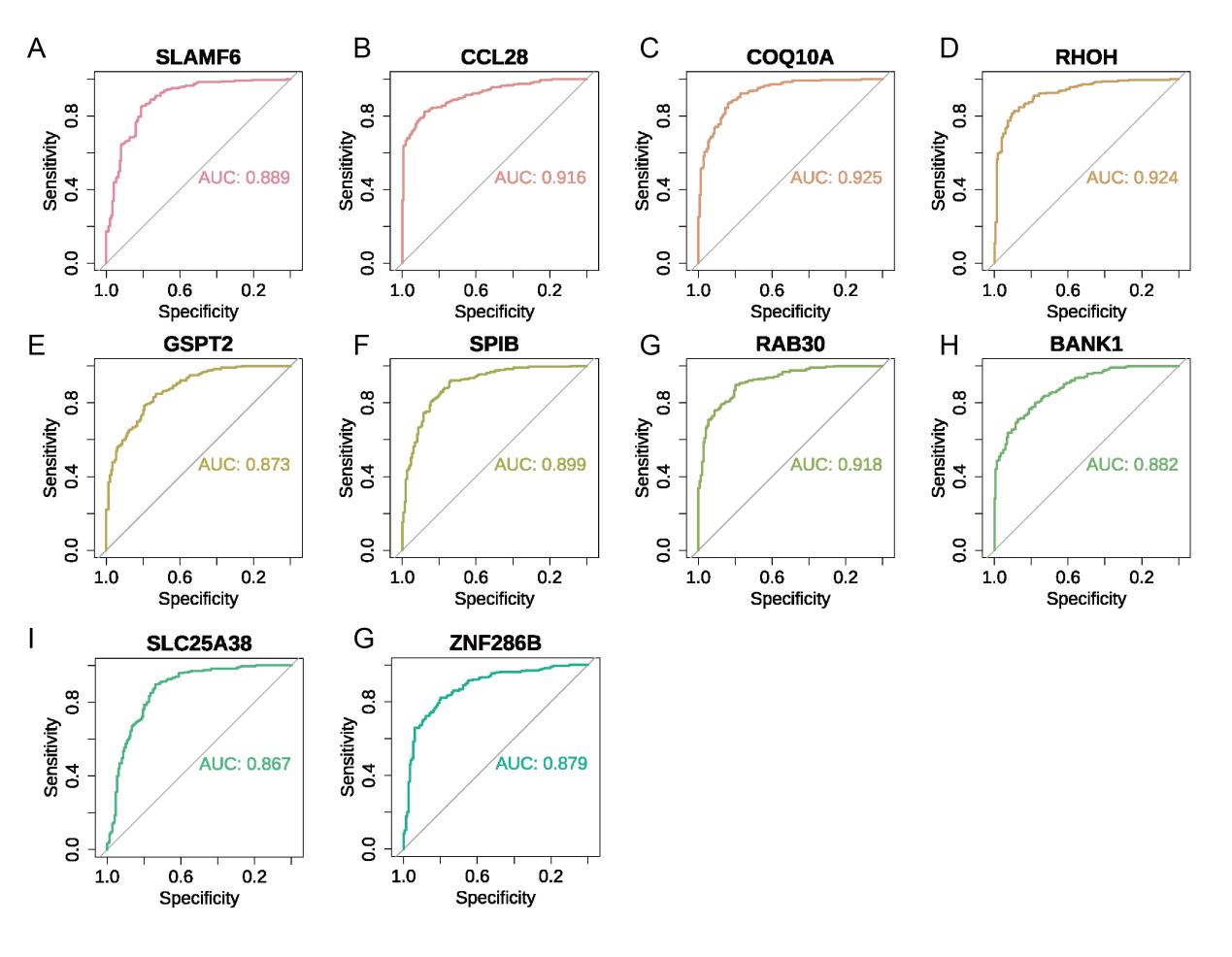


**Figure S8. ROC curve shows the classification efficiency of 10 marker genes.**


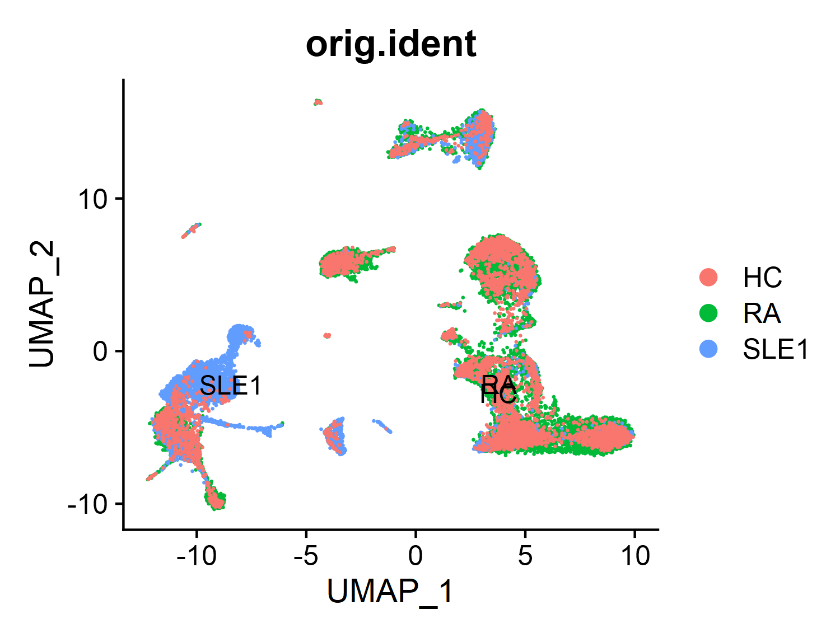


**Figure S9.** **UMAP Clustering Across Different Samples.**

1. **Relationship of marker genes to immune response and immune diseases.**

*SLAMF6* (signaling lymphocytic activation molecule family member 6) is a transmembrane protein that plays a critical role in immune regulation. Multiple studies have shown that *SLAMF6* is involved in the interactions between immune cells. In patients with RA, the expression level of *SLAMF6* is significantly elevated and positively correlated with disease activity and severity. Researchers have also identified a functional variant of *SLAMF6* gene (rs148363003) that is associated with susceptibility to and severity of RA. Moreover, inhibition of the *SLAMF6* signaling pathway has been shown to alleviate arthritis symptoms[[17](#_ENREF_17)]. These results suggest that *SLAMF6* plays an important role in the pathophysiological processes of RA and is considered a potential therapeutic target.

*CCL28* (chemokine (C-C motif) ligand 28), also known as mucosa-associated epithelial chemokine (MEC), is a chemokine that exerts its effects by regulating the chemotaxis of cells expressing the chemokine receptors *CCR3* and *CCR10*. One study has shown that *CCL28* and its receptor *CCR10* play an important role in the pathogenesis of RA. The researchers evaluated the expression of *CCL28* and *CCR10* in RA synovial tissue or fluid by histology or enzyme-linked immunosorbent assay (ELISA). They found that the expression levels of *CCL28* and *CCR10* were significantly increased in RA synovial tissue, mainly co-expressed in RA myeloid cells and endothelial cells. In addition, *CCL28* and *CCR10* promote monocyte migration in RA. Therefore, the *CCL28-CCR10* pathway may be involved in the recruitment of monocytes in RA joints, promoting synovial inflammation and bone destruction[[18](#_ENREF_18)].

The *BANK1* gene encodes the *BANK1* protein, which is an important B cell signaling molecule. Research has shown that the *BANK1* gene is associated with the risk of systemic lupus erythematosus (SLE) and affects peripheral B cell signaling and development. The article suggests that the SLE risk variants of the *BANK1* gene are associated with the incidence of SLE and can cause abnormalities in peripheral B cell signaling pathways, thereby affecting B cell development and function. Through the study of human peripheral blood B cells, it has been found that these variants can affect the expression of multiple signaling pathways and cytokines, thus affecting B cell activation, proliferation, and differentiation. The results indicate that the SLE susceptibility variants of the *BANK1* gene may promote the development of lupus by altering B cell signaling, increasing the level of *FOXO1* protein, and enhancing memory B cell development[[19](#_ENREF_19)].

Research has also shown that *BANK1* is a susceptibility gene for rheumatoid arthritis (RA), providing the first evidence of epistasis between *BANK1* and *BLK* in RA. An epistatic interaction was detected between *BANK1* rs3733197 and BLK rs13277113 (p-interaction=0.037). The presence of the *BANK1* rs3733197 G allele increased the risk of RA in individuals carrying the *BLK* rs13277113 GG genotype, suggesting that *BANK1* and *BLK* may play a role in the pathogenesis of RA. Overall, abnormal expression of the *BANK1* gene in RA and SLE may be involved in the development of these diseases through the regulation of B cell signaling pathways and autoantigen presentation mechanisms[[20](#_ENREF_20)].

Therefore, the expression of these 10 gene markers may distinguish the infection source of RA and SLE patients, and help clinicians use Antiviral drug or antibiotics to achieve personalized treatment of autoimmune diseases.

**Reference**

1. Binvignat M, Miao BY, Wibrand C, Yang MM, Rychkov D, Flynn E, Nititham J, Tamaki W, Khan U, Carvidi A *et al*: **Single-cell RNA-Seq analysis reveals cell subsets and gene signatures associated with rheumatoid arthritis disease activity**. *JCI insight* 2024, **9**(16).

2. Satija R, Farrell JA, Gennert D, Schier AF, Regev A: **Spatial reconstruction of single-cell gene expression data**. *Nat Biotechnol* 2015, **33**(5):495-502.

3. Zhang X, Lan Y, Xu J, Quan F, Zhao E, Deng C, Luo T, Xu L, Liao G, Yan M *et al*: **CellMarker: a manually curated resource of cell markers in human and mouse**. *Nucleic Acids Res* 2019, **47**(D1):D721-D728.

4. Irizarry RA, Hobbs B, Collin F, Beazer-Barclay YD, Antonellis KJ, Scherf U, Speed TP: **Exploration, normalization, and summaries of high density oligonucleotide array probe level data**. *Biostatistics* 2003, **4**(2):249-264.

5. Jaffe AE, Irizarry RA: **Accounting for cellular heterogeneity is critical in epigenome-wide association studies**. *Genome Biol* 2014, **15**(2):R31.

6. Wilkerson MD, Hayes DN: **ConsensusClusterPlus: a class discovery tool with confidence assessments and item tracking**. *Bioinformatics* 2010, **26**(12):1572-1573.

7. Barbie DA, Tamayo P, Boehm JS, Kim SY, Moody SE, Dunn IF, Schinzel AC, Sandy P, Meylan E, Scholl C *et al*: **Systematic RNA interference reveals that oncogenic KRAS-driven cancers require TBK1**. *Nature* 2009, **462**(7269):108-112.

8. Charoentong P, Finotello F, Angelova M, Mayer C, Efremova M, Rieder D, Hackl H, Trajanoski Z: **Pan-cancer Immunogenomic Analyses Reveal Genotype-Immunophenotype Relationships and Predictors of Response to Checkpoint Blockade**. *Cell Rep* 2017, **18**(1):248-262.

9. Newman AM, Liu CL, Green MR, Gentles AJ, Feng W, Xu Y, Hoang CD, Diehn M, Alizadeh AA: **Robust enumeration of cell subsets from tissue expression profiles**. *Nat Methods* 2015, **12**(5):453-457.

10. Langfelder P, Horvath S: **WGCNA: an R package for weighted correlation network analysis**. *BMC bioinformatics* 2008, **9**:559.

11. Kerr MK: **Linear models for microarray data analysis: hidden similarities and differences**. *J Comput Biol* 2003, **10**(6):891-901.

12. Ashburner M, Ball CA, Blake JA, Botstein D, Butler H, Cherry JM, Davis AP, Dolinski K, Dwight SS, Eppig JT *et al*: **Gene ontology: tool for the unification of biology. The Gene Ontology Consortium**. *Nat Genet* 2000, **25**(1):25-29.

13. Liberzon A, Subramanian A, Pinchback R, Thorvaldsdottir H, Tamayo P, Mesirov JP: **Molecular signatures database (MSigDB) 3.0**. *Bioinformatics* 2011, **27**(12):1739-1740.

14. Subramanian A, Tamayo P, Mootha VK, Mukherjee S, Ebert BL, Gillette MA, Paulovich A, Pomeroy SL, Golub TR, Lander ES *et al*: **Gene set enrichment analysis: A knowledge-based approach for interpreting genome-wide expression profiles**. *Proceedings of the National Academy of Sciences* 2005, **102**(43):15545-15550.

15. Chen H, Kong Y, Yao Q, Zhang X, Fu Y, Li J, Liu C, Wang Z: **Three hypomethylated genes were associated with poor overall survival in pancreatic cancer patients**. *Aging (Albany NY)* 2019, **11**(3):885-897.

16. Lee YW, Choi JW, Shin E-H: **Machine learning model for predicting malaria using clinical information**. *Computers in Biology and Medicine* 2021, **129**.

17. Xia G, Li Y, Pan W, Qian C, Ma L, Zhou J, Xu H, Cheng C: **SLAMF6 is associated with the susceptibility and severity of rheumatoid arthritis in the Chinese population**. *Journal of orthopaedic surgery and research* 2022, **17**(1):13.

18. Chen Z, Kim SJ, Essani AB, Volin MV, Vila OM, Swedler W, Arami S, Volkov S, Sardin LV, Sweiss N *et al*: **Characterising the expression and function of CCL28 and its corresponding receptor, CCR10, in RA pathogenesis**. *Annals of the rheumatic diseases* 2015, **74**(10):1898-1906.

19. Dam EM, Habib T, Chen J, Funk A, Glukhova V, Davis-Pickett M, Wei S, James R, Buckner JH, Cerosaletti K: **The BANK1 SLE-risk variants are associated with alterations in peripheral B cell signaling and development in humans**. *Clinical immunology* 2016, **173**:171-180.

20. Genin E, Coustet B, Allanore Y, Ito I, Teruel M, Constantin A, Schaeverbeke T, Ruyssen-Witrand A, Tohma S, Cantagrel A *et al*: **Epistatic interaction between BANK1 and BLK in rheumatoid arthritis: results from a large trans-ethnic meta-analysis**. *PloS one* 2013, **8**(4):e61044.
